# Supplementary material for: Temperature-dependent modulation of aberrant influenza A virus RNA synthesis affects innate immune signaling
Source: bioRxiv. 2025 May 22:2025.05.19.654939. Preprint. [Version 2] doi: 10.1101/2025.05.19.654939 (PMC12139806; doi:10.1101/2025.05.19.654939)
Supplement: Supplementary file 1 [file NIHPP2025.05.19.654939v2-supplement-1.pdf]

bioRxiv preprint doi: https://doi.org/10.1101/2025.05.19.654939; this version posted May 22, 2025. The copyright holder for this preprint (which was not certified by peer review) is the author/funder, who has granted bioRxiv a license to display the preprint in perpetuity. It is made available under aCC-BY 4.0 International license.

Supplemental information

Temperature-dependent modulation of aberrant influenza A virus RNA synthesis affects innate immune signaling

Karishma Bisht[1*], Daniel R. Weilandt[2,3,4], Caitlin H. Lamb[1], Elizaveta Elshina[1], Cameron Myhrvold[1,2,5,6], Aartjan J.W. te Velthuis[1*]

[1] Lewis Thomas Laboratory, Department of Molecular Biology, Princeton University, 08544 New Jersey, USA.
[2] Department of Chemistry, Princeton University, Princeton, NJ, USA.
[3] Lewis-Sigler Institute of Integrative Genomics, Princeton University, Princeton, NJ, USA.
[4] Ludwig Institute for Cancer Research, Princeton University, Princeton, NJ, USA.
[5] Department of Chemical and Biological Engineering, Princeton University, Princeton, NJ 08544, USA.
[6] Omenn-Darling Bioengineering Institute, Princeton University, Princeton, NJ 08544, USA.

* Address correspondence to karishma.bisht@princeton.edu or aj.te.velthuis@princeton.edu

bioRxiv preprint doi: https://doi.org/10.1101/2025.05.19.654939; this version posted May 22, 2025. The copyright holder for this preprint (which was not certified by peer review) is the author/funder, who has granted bioRxiv a license to display the preprint in perpetuity. It is made available under aCC-BY 4.0 International license.

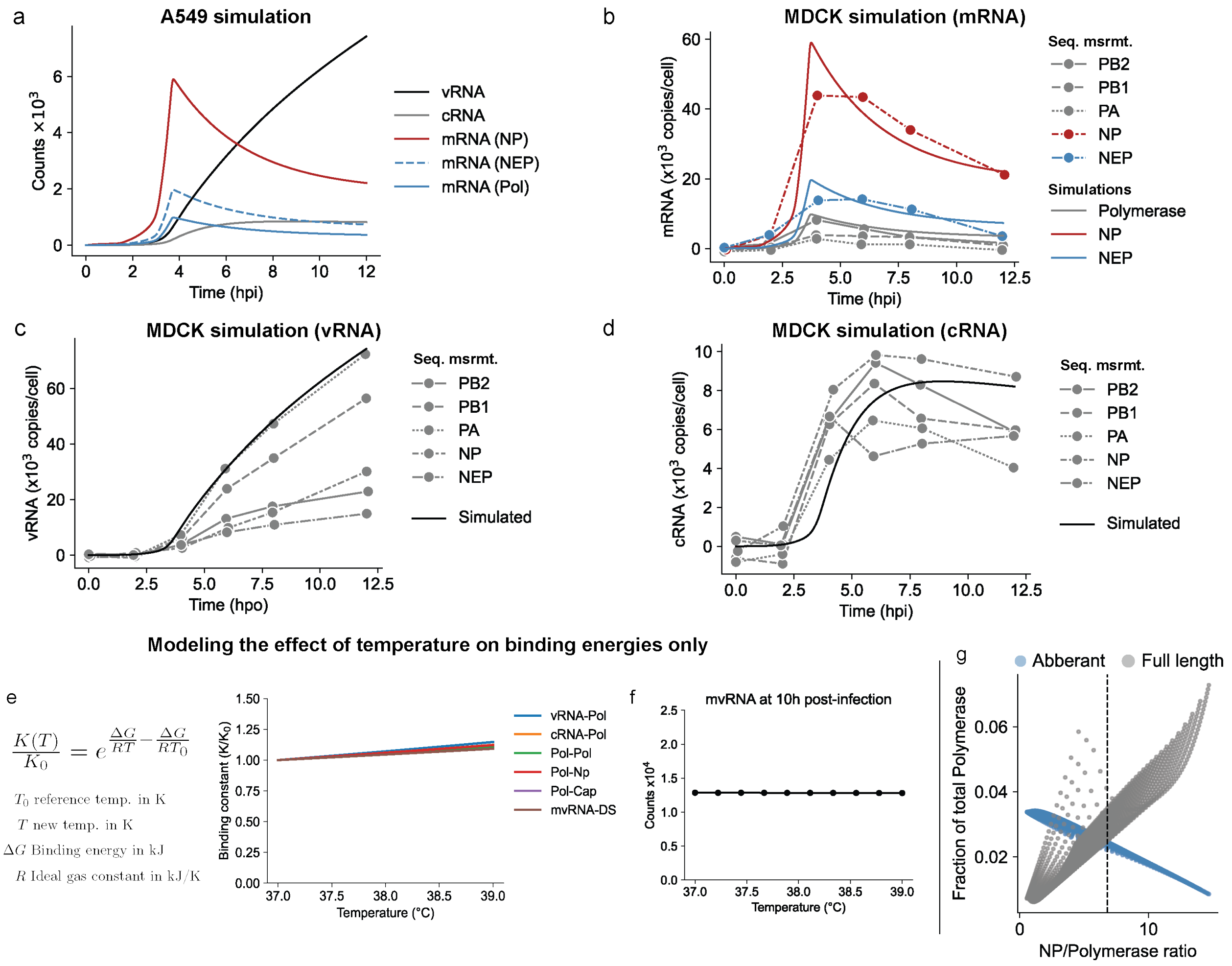

Supplementary Fig.1| **a,** Model prediction of cRNA, vRNA and mRNA subspecies for single infection cycle of IAV in A549 cell line. Note that we assume that the replication of the eight IAV segments (vRNA and cRNA) is not fundamentally different, and that the expression differences among the segments are driven by variations in transcription (mRNA) efficiency, which is why only one vRNA and cRNA are simulated here. Comparing the model predictions to experimental data collected in MDCK cells for **b,** mRNA levels for polymerase segments, nucleoprotein and nuclear exit protein, **c,** vRNA level, **d,** cRNA level. Model predictions for temperature effects on only binding constant **e,** Mathematical expression and simulation for temperature variation on binding constants. **f,** mvRNA level as function of the temperature effect on binding constants only. **g,** Fraction of polymerase engaged in transcription of aberrant and full-length products as a function of NP/polymerase ratio.

bioRxiv preprint doi: https://doi.org/10.1101/2025.05.19.654939; this version posted May 22, 2025. The copyright holder for this preprint (which was not certified by peer review) is the author/funder, who has granted bioRxiv a license to display the preprint in perpetuity. It is made available under aCC-BY 4.0 International license.

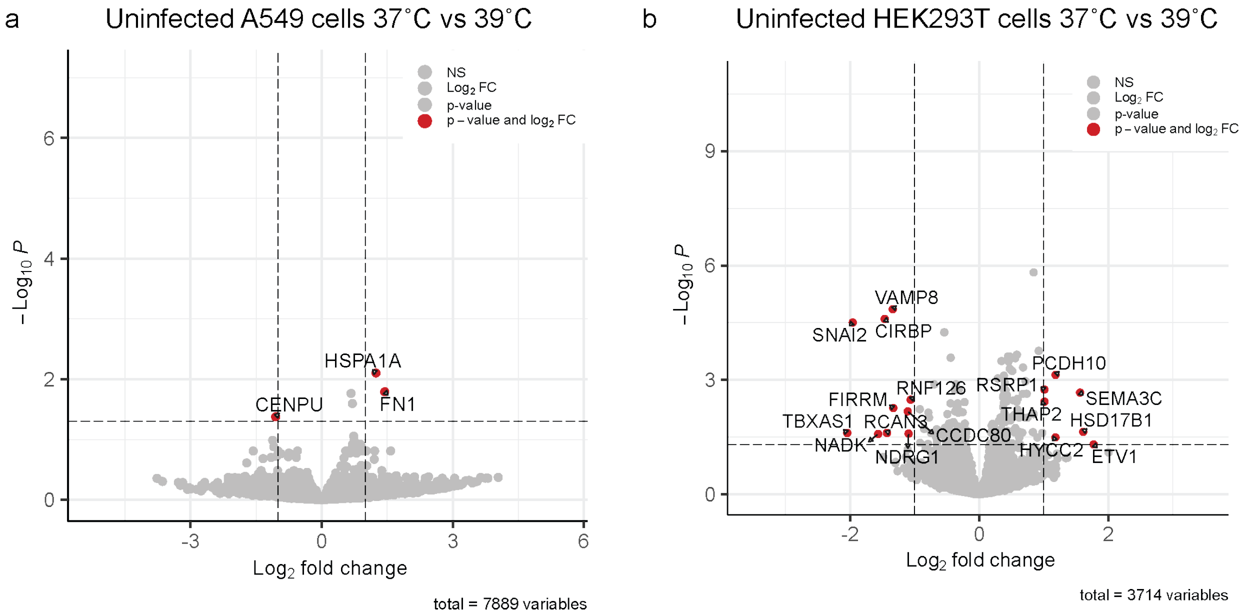

Supplementary Fig.2| **a,** Volcano plot illustrating differentially expressed genes in A549 mock infected cells after being adapted to 37˚C and 39˚C for 3 days **b,** Volcano plot illustrating the differentially expressed genes in HEK293T cells after being adapted to 37˚C and 39˚C for 3 days. RNA-seq was performed on 3 biological replicates.

bioRxiv preprint doi: https://doi.org/10.1101/2025.05.19.654939; this version posted May 22, 2025. The copyright holder for this preprint (which was not certified by peer review) is the author/funder, who has granted bioRxiv a license to display the preprint in perpetuity. It is made available under aCC-BY 4.0 International license.

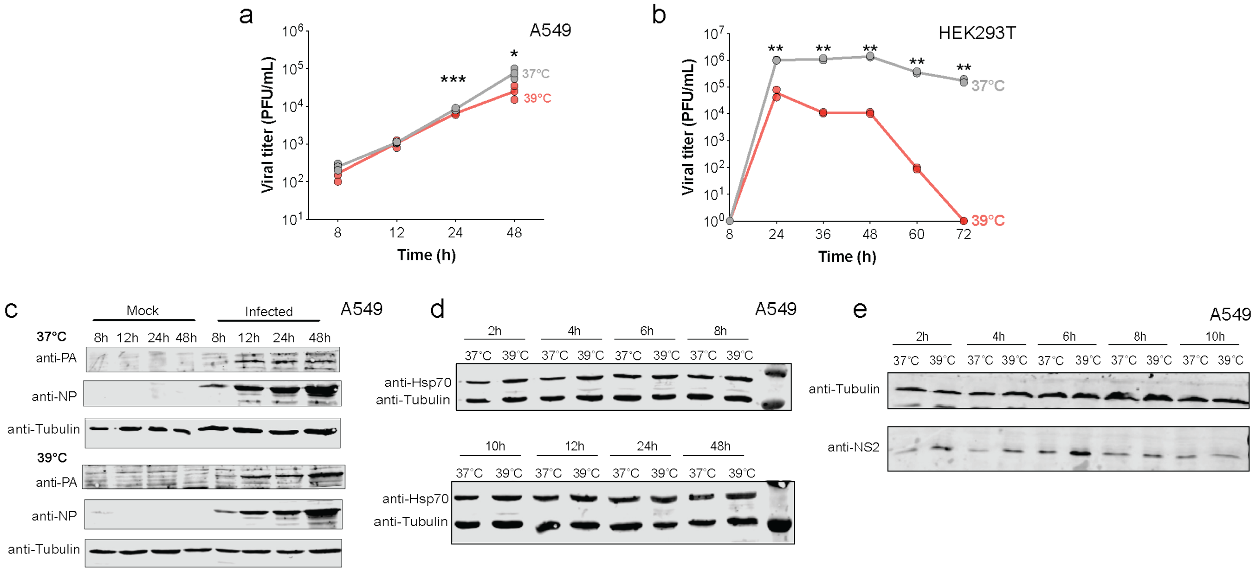

Supplementary Fig.3| Growth kinetics of lab adapted WSN virus in A549 (**a**) and HEK293T (**b**) cells at different temperatures (37°C, or 39°C). A549 cells or HEK293T cells were infected at an MOI of 0.01 at different temperatures 37°C and 39°C. The supernatants of the infected cells were harvested at the indicated times, and the virus titers were determined by performing plaque assays in MDCK cells at 37°C. Data are shown as the mean of triplicate experiments. Error bars indicate the standard deviation. The P values were determined by using an unpaired t test. (*$P < 0.05$; **$P < 0.01$; ***$P < 0.001$). **c,d,e,** Cell lysates were collected at different time points from infected and mock cells and analyzed by immunoblotting with indicated antibodies.

bioRxiv preprint doi: https://doi.org/10.1101/2025.05.19.654939; this version posted May 22, 2025. The copyright holder for this preprint (which was not certified by peer review) is the author/funder, who has granted bioRxiv a license to display the preprint in perpetuity. It is made available under aCC-BY 4.0 International license.

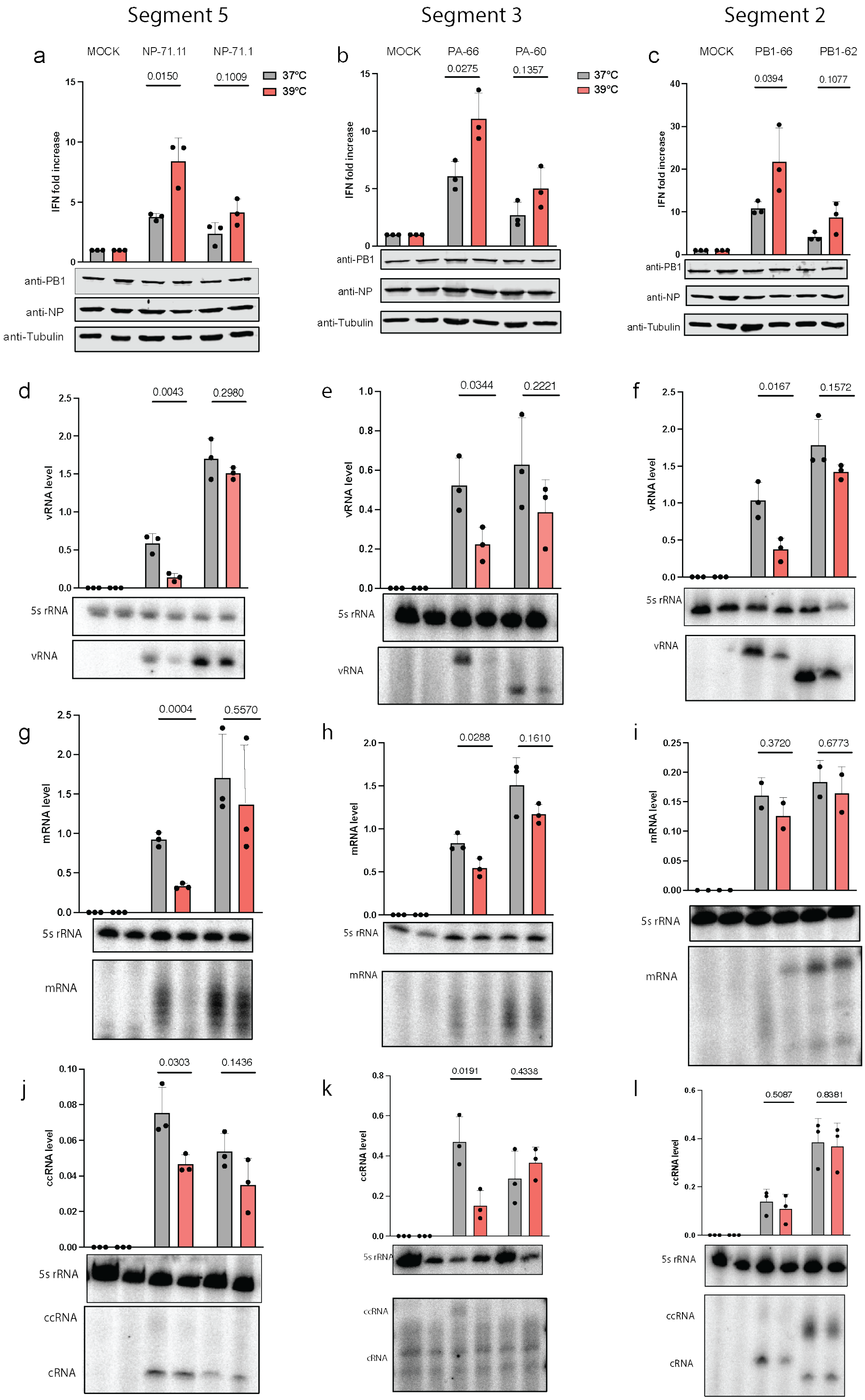

bioRxiv preprint doi: https://doi.org/10.1101/2025.05.19.654939; this version posted May 22, 2025. The copyright holder for this preprint (which was not certified by peer review) is the author/funder, who has granted bioRxiv a license to display the preprint in perpetuity. It is made available under aCC-BY 4.0 International license.

Supplementary Fig.4| a,b,c, Analysis of IFN-β promoter activity induced by the replication of segment 5, 3 or 2 mvRNAs by the WSN RNA polymerase. PB1, NP and tubulin expression was analysed by western blot. *P* values were determined using a two-sided, unpaired *t*-test. **d,e,f,** Steady state mvRNA and 5S rRNA levels measured and quantified, 24 hours post-transfection by primer extension in the presence of WSN RNA polymerase. **g,h,I**, Steady state mRNA and 5S rRNA levels measured and quantified, 24 hours post-transfection by primer extension in the presence of WSN RNA polymerase. **j,k,l**, Steady state ccRNA levels measured and quantified, 24 hours post-transfection by primer extension in the presence of WSN RNA polymerase. Data are shown as the mean of triplicate experiments. Error bars indicate the standard deviation. The *P* values were determined by using an unpaired *t* test.

bioRxiv preprint doi: https://doi.org/10.1101/2025.05.19.654939; this version posted May 22, 2025. The copyright holder for this preprint (which was not certified by peer review) is the author/funder, who has granted bioRxiv a license to display the preprint in perpetuity. It is made available under aCC-BY 4.0 International license.

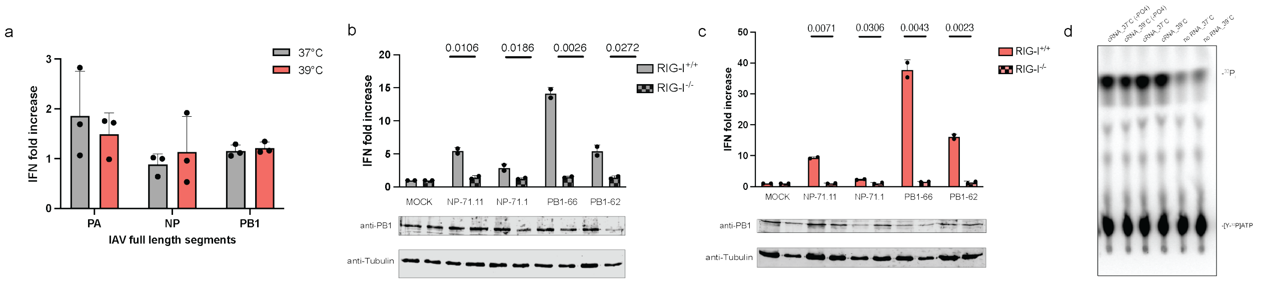

Supplementary Fig.5| **a,** IFN-β promoter activity induced by the expression of full-length segment 3, 5 and 2 in HEK293T cells adapted at 37˚C and 39˚C. **b,c,** IFN-β promoter activity induced by expression of segment 5 and 2 mvRNAs in wild-type (RIG-I$^{+/+}$) or RIG-I knockout (RIG-I$^{-/-}$) HEK293 cells at the two temperatures. Bottom two panels show western blot analysis**.** Data are shown as the mean of two independent experiments **d,** ATPase activity of recombinant RIG-I was assessed in the presence of *in vitro* transcribed PA66-based cRNA, and dephosphorylated cRNA, as well as no RNA control. Data are shown as the mean of triplicate experiments. Error bars indicate the standard deviation. The *P* values were determined by using an unpaired *t* test.

bioRxiv preprint doi: https://doi.org/10.1101/2025.05.19.654939; this version posted May 22, 2025. The copyright holder for this preprint (which was not certified by peer review) is the author/funder, who has granted bioRxiv a license to display the preprint in perpetuity. It is made available under aCC-BY 4.0 International license.

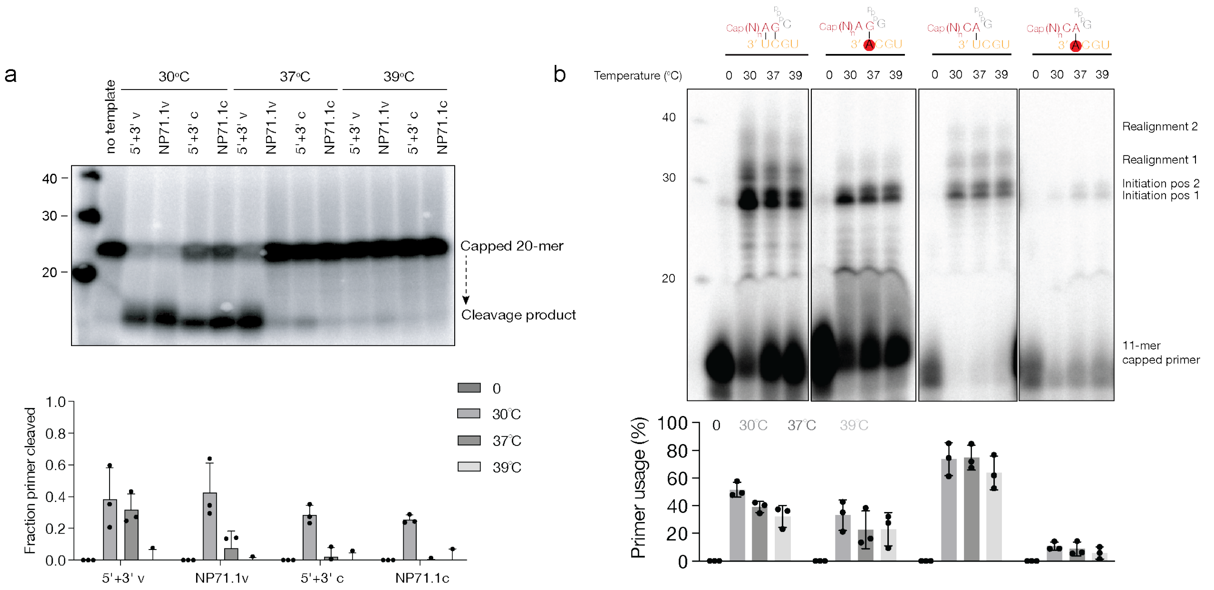

Supplementary Fig.6| **a,** Cap-snatching of a ^{32}P-labeled capped, 20-nt long RNA primer by the IAV RNA polymerase at 30˚C, 37˚C and 39˚C. Cleavage reactions were analyzed by denaturing PAGE. Lower panel shows fraction of the primer cleaved at three temperatures. **b,** Extension of a radiolabeled, capped, 11-nucleotide-long RNA primer ending in 3′ AG on the wild-type or 3′ 1U→A vRNA promoter and 3′ CA on the wild-type or 3′ 1U→A vRNA promoter, at 30˚C, 37˚C and 39˚C. Quantitation of data in the lower panel shows percentage of primer used at the three temperatures.

bioRxiv preprint doi: https://doi.org/10.1101/2025.05.19.654939; this version posted May 22, 2025. The copyright holder for this preprint (which was not certified by peer review) is the author/funder, who has granted bioRxiv a license to display the preprint in perpetuity. It is made available under aCC-BY 4.0 International license.

Table S1: Model parameters at 37 and 39˚C.

Symbol	Description	Source	Value 37˚C	Value 39˚C	Unit
k	Universal diffusion limited binding constant	36	2.78×10^5	2.78×10^5	1/h/M
$K_{vRNA/Pol}$	Binding constant of vRNA to polymerase complex	22	0.5	0.57	nM
$K_{cRNA,Pol}$	Binding constant of cRNA to polymerase complex	23	13	14.6	nM
$K_{Pol,Pol}$	Binding constant two polymerase complexes	assumption	100	101	nM
$K_{Pol,NP}$	Binding constant polymerase to first nucleoprotein	24,25,26	15	16.8	nM
$K_{Pol,Cap}$	Binding constant polymerase to host mRNA cap	27	1	1.1	μM
V_{cap}	Synthesis rate for caps	methods	10^3	10^3	#CAP/h
$k_{d,cap}$	Degradation rate for caps	assumption	0.1	0.1	1/h
$k_{t,NEP}$	Transcription rate of nuclear export protein	21*	35	14	1/h
$k_{t,Pol}$	Transcription rate of polymerase mRNA	21*	17.5	14	1/h
$k_{t,NP}$	Transcription rate of nucleoprotein	21*	105	42	1/h
$k_{t,mini}$	Transcription rate of mvRNA	21*	3.5	3.5	1/h
k_{tl}	Universal translation rate	21	6	6	1/h
k_{rv}	Replication rate of vRNA (from cRNA)	21	13.85	13.85	1/h
k_{rc}	Replication rate of cRNA (from vRNA)	21	1.38	1.38	1/h
$k_{r,mini}$	Initiation rate of mvRNA rate (from vRNA)	assumption	1.38	1.38	1/h
k_d	Degradation rate of mRNA	21	0.33	0.33	1/h
$k_{d,vc}$	Degradation rate of nascent cRNA and vRNA	21	36	36	1/h
$k_{d,RNP}$	Degradation rate of RNPs	21	0.09	0.09	1/h
$k_{d,p}$	Degradation rate of proteins	21	0.5	0.5	1/h
k_{exp}	Export rate of RNPs per NEP	21	10^{-8}	10^{-8}	1/h/#NEP
z	Stoichiometry of nucleoprotein per RNP	12	20	20	#NP/#POL
V_r	Viral replication volume in nucleus	methods	10^{-15}	10^{-15}	L

*Computed from the transcription rate per nucleotide reported.

bioRxiv preprint doi: https://doi.org/10.1101/2025.05.19.654939; this version posted May 22, 2025. The copyright holder for this preprint (which was not certified by peer review) is the author/funder, who has granted bioRxiv a license to display the preprint in perpetuity. It is made available under aCC-BY 4.0 International license.

Table S2: Model species

Symbol	Description
$[CAP]$	Pools of CAP sequences in the vicinity of the RNP
$[S_v]$	Representative RNP with viral RNA*
$[S_c]$	Representative RNP with complementary RNA*
$[S_v]_{cyt}$	Exported representative RNP with viral RNA* (cytosolic)
$[NP]$	Nucleoproteins
$[NEP]$	Nuclear exit protein
$[Pol]$	Polymerase protein**
$[mRNA_{NP}]$	mRNA encoding nucleoproteins
$[mRNA_{NEP}]$	mRNA encoding nuclear export protein
$[mRNA_{Pol}]$	mRNA encoding RNA polymerase protein subunit**
$[S_v, Pol]$	Representative vRNP polymerase complex*,**
$[S_c, Pol]$	Representative cRNP polymerase complex*,**
$[S_v, Pol, NP]$	Representative vRNP polymerase complex*,**
$[S_c, Pol, NP]$	Representative cRNP polymerase complex*,**
$[mcRNA]$	Mini viral RNA positive sense
$[mvRNA]$	Mini viral RNA negative sense
$[mcRNA, Pol]$	Mini viral RNA positive sense polymerase complex**
$[mvRNA, Pol]$	Mini viral RNA negative sense polymerase complex**
$[mcRNA, Pol, Pol]$	Mini viral RNA positive sense two polymerase complex**
$[mvRNA, Pol, Pol]$	Mini viral RNA negative sense two polymerase complex**
$[cap.mcRNA]$	Capped mini viral RNA positive sense
$[cap.mvRNA]$	Capped mini viral RNA negative sense
$[ds.mvRNA]$	Double stranded mini viral RNA

* Representative RNP meaning average across all 8 segments

** Polymerase protein, meaning sum across the 3 polymerase subunits

bioRxiv preprint doi: https://doi.org/10.1101/2025.05.19.654939; this version posted May 22, 2025. The copyright holder for this preprint (which was not certified by peer review) is the author/funder, who has granted bioRxiv a license to display the preprint in perpetuity. It is made available under aCC-BY 4.0 International license.

Table S3

Template Name	Sequence of the RNA templates (5′ to 3′)
NP STOP	WSN NP full-length STOP (Start codon ATG mutated in stop codon TCG)
PA	WSN PA full length
PB1 STOP	WSN PB1 full length STOP (Start codon ATG mutated in stop codon TCG)
NP246	AGUAGAAACAAGGGUAUUUUUCUUUAAUUGUCGUACUCCUCUGCAUUGUCUCCGAAG AAAUAAGAUCCUUCAUUACUCAUGUCAAAGGAGGGCACGAUCGGGCUCGUUGCCUUU UCGUCCGAGAGCUCGAAGACUCCCCGCCCCUGGAAAGACACUAGUCUCCAUCUGUUC GUAAGAUCGUUUGGUGCCUUUGGUCGCCAUGAUUUCGAUGUCACUCUGUACUAGUC UACCCUGCUUUUGCU
NP71.1	AGUAGAAACAAGGGUAUUUUUCUUUACUAGUUAGGUAGUAUACCUAGUAACUAGUCU ACCCUGCUUUUGCU
NP71.11	AGUAGAAACAAGGGUAUUUUUCUUUACUAGUGGCAGCAAAAGCACCCAUACUAGUCU ACCCUGCUUUUGCU
PA-60	AGUAGAAACAAGGUACUUUUUUGGACAGUAUGCCAUUUUGAAUCAGUACCUGCUUUC GCU
PA-66	AGUAGAAACAAGGUACUUUUUUGGACAGUAUGGAUAGCACAUUUUGAAUCAGUACCU GCUUUCGCU
PB1-66	AGUAGAAACAAGGCAUUUUUUCAUGAAGGACAAGCUAAACAUUCAAAUGGUUUGCCU GCUUUCGCU
PB1-62	AGUAGAAACAAGGCAUUUUAAGUCGGAUUGACAUCCAUUCAAAUGGUUUGCCUGCUU UCGCU
5S 100	TCCCAGGCGGTCTCCCATCC
cRNA 3’	GGCCUUGUUUCUACU
cRNA 5’	AGCGAAAGCAGGCC
vRNA 3’	GGCCUGCUUUUGCU
vRNA 5’	AGUAGUAACAAGGCC

bioRxiv preprint doi: https://doi.org/10.1101/2025.05.19.654939; this version posted May 22, 2025. The copyright holder for this preprint (which was not certified by peer review) is the author/funder, who has granted bioRxiv a license to display the preprint in perpetuity. It is made available under aCC-BY 4.0 International license.

Table S4: DNA oligonucleotides used for primer extension.

Primer name	Target RNA	DNA oligonucleotide (5' to 3')
NP-	Non-full-length NP vRNA and aberrant products	AGCAAAAGCAGGGTAGACTAGT
NP+	Non-full-length NP mRNA	ACTAGTCTACCCTGCTTTTGC
NP5'	Non-full-length NP cRNA	AGTAGAAAACAAGGGTATTTTTC
PA_PEplus2	Non-full-length PA cRNA	AGTAGAAACAAGGTACTTTTTTGGACA
PA vRNA	Non-full-length PA vRNA and aberrant products	AGCGAAAGCAGGTACTGATTC
PA_PEplus_short	Non-full-length PA mRNA	TTTTGAATCAGTACCTGCTTTCG
PB1-3'	Non-full-length PB1 vRNA	AGCGAAAGCAGGCAAACCATTTG
PB1 cRNA	Non-full-length PB1 cRNA and aberrant products	AGTAGAAACAAGGCATTTT
PB1_PEplus_short	Non-full-length PB1 mRNA	CAAATGGTTTGCCTGCTTTCG
PA-2119	Full length PA vRNA	GGGTTTTGCCTGCTTTCG
PA-135	Full length PA cRNA and mRNA	TGCTGCAAATTTGTTTGTTTCG
NP149-	Full length NP vRNA	ATTTCTTCGGAGACAATGCAG
NP149+	Full length NP cRNA and mRNA	TAAGATCGTTTGGTGCCTTTG
5S_100	5S sRNA; loading control	TCCCAGGCGGTCTCCCATCC

bioRxiv preprint doi: https://doi.org/10.1101/2025.05.19.654939; this version posted May 22, 2025. The copyright holder for this preprint (which was not certified by peer review) is the author/funder, who has granted bioRxiv a license to display the preprint in perpetuity. It is made available under aCC-BY 4.0 International license.

Table S5: Antibody used for western blot.

Primary antibody		
NP	Rabbit, GTX125989, GeneTex	1:2000
PB1	Rabbit, GTX125923, GeneTex	1:1000
PB2	Rabbit, GTX125926, GeneTex	1:1000
PA	Rabbit, GTX125932, GeneTex	1:1000
Hsp70	Rabbit, 24532, Cayman Chemicals	1:500
NS1	Rabbit, PA5-32243, Invitrogen	1:1000
γ-tubulin	Rat, MCA77G, Bio-Rad	1:5000
Secondary antibody		
IRDye 680 goat anti-rat	926-68076, LI-COR	1:10000
IRDye 800 donkey anti-rabbit	926-32213, LI-COR	1:10000
